# Acne keloidalis nuchae and hypertension in black subjects: a case–control study

**DOI:** 10.1186/s13104-020-05274-0

**Published:** 2020-09-14

**Authors:** Bayaki Saka, Julienne Noude Teclessou, Sefako Abla Akakpo, Soulemane Pessinaba, Piham Gnossike, Garba Mahamadou, Panawé Kassang, Abas Mouhari-Toure, Koussake Kombate, Palokinam Pitché

**Affiliations:** 1 Service de Dermatolgie et IST, CHU Sylvanus Olympio, Université de Lomé, BP. 30785, Lome, Togo; 2 Service de Dermatolgie et IST, CHU Campus Université de Lomé, Lome, Togo; 3 Service de Cardiologie, CHU Campus, Université de Lomé, Lome, Togo; 4 Centre National de Dermatologie de Gbossimé, Lome, Togo; 5 Service de dermatolgie et IST, CHU Kara, Université de Kara, Kara, Togo

**Keywords:** AKN, Hypertension, Black subject

## Abstract

**Objective:**

The aim of this case–control study was to look for an association between hypertension and acne keloidalis nuchae (AKN) in black subjects.

**Results:**

We recruited 303 consenting subjects comprising 101 patients with AKN and 202 controls, case-matched by age (± 5 years). The mean patients age was 34.9 ± 10.7 years versus 35.6 ± 11.2 years for controls. The average duration of AKN progression in cases prior to consultation was 1831 days (5 years). The most frequently observed AKN lesions were papules (73/101; 72.3%), fibrous scars (42/101; 41.6%) and folliculitis/pustules (41/101; 40.6%). In terms of quality of life, the mean score of dermatology life quality index was 8.3 ± 5.2 (extremes: 0 to 22). In multivariate analysis, having a BMI of 25 kg/m^2^ or more (OR = 4.91; p < 0.001) and having systolic hypertension (OR = 1.22; p = 0.010) were associated with AKN.

## Introduction

Acne keloidalis nuchae (AKN) is a chronic scarring folliculitis observed mainly in men of African descent [1, 2]. Contributing factors appear to be varied and involve androgens, inflammation, infections, trauma, genetics and ingrown hairs [3]. Piecemeal data suggest a possible relationship between hypertension and AKN [1, 4]. Verma and Wollina already reported one case of AKN associated with hypertension in 2010 [4]. In addition, hypertensive patients are 6.75 times more likely to have extraoccipital/extra-nuchal lesions than non-hypertensive patients [1]. Given that AKN mainly affects men of African descent [1, 2], and that hypertension is more frequent and more severe in black subjects than in Caucasian subjects [5], is there a link between hypertension and AKN? We therefore conducted a case–control study to look for a possible association between hypertension and AKN in the black subject.

## Main text

### Method

We conducted a case–control study from January to December 2018 in three dermatology departments (CHU Sylvanus Olympio, CHU campus and Gbossimé centre) in Lomé, Togo. Cases were male patients, over 18 years of age, seen in dermatology for AKN located at the neck and/or extending to other parts of the scalp. For the controls, any lesion suggestive of AKN (papular, pustular, hypertrophic or keloid lesion) of the neck was a non-inclusion criteria. Each case was matched with two controls per age (± 5 years). Cases were recruited at outpatients dermatological clinics and controls recruited at outpatients dermatological clinics and then in other hospital departments. The recruitment was consecutive and comprehensive. The questionnaire used for general and dermatological examination was developped for this study (Additional file 1), and was the same for both cases and controls, except for the part concerning AKN. Each case and control was subjected to a general examination (including blood pressure measurement) and dermatological examination. Blood pressure was taken using a cuff and needle manometer by auscultatory method on a seated subject. We defined systolic hypertension as systolic blood pressure ≥ 140 mmHg and diastolic hypertension as diastolic blood pressure ≥ 90 mmHg at two separate consecutive visits, two weeks apart. For defining hypertension, participants were brought back for a second visit to the clinic (either for derm follow-up or specifically for blood pressure measurement). In both cases and controls, we excluded known hypertensive subjects under treatment, even if, blood pressure is normal (because if they're on antihypertensive drugs, it's because they have hypertension, and that would be a selection bias). We also did not include patients undergoing treatment (history of intra-leaved corticosteroid injections) which could influence blood pressure. Also, between the first and second visit, topical steroids or IL steroids to treat AKN were not used in order to not affect blood pressure. For cases, in addition to the general and dermatological examination, quality of life (QOL) was assessed according to the Dermatology Life Quality Index (DLQI) [6], with a total score between 0 and 30. A DLQI score less than or equal to 1 reflected no effect of AKN on the patient's QOL, a score between 2 and 5 reflected a small effect, a score between 6 and 10 reflected a moderate effect, a score between 11 and 20 reflected a large effect, and finally a score between 21 and 30 reflected an extremely large effect on the patient's QOL.

#### Statistical analysis

The questionnaires were entered using EPIDATA software version 3.1. Descriptive statistics were conducted and the results presented in the form of frequency and percentage tables for the qualitative variables. Quantitative variables were presented as means (± standard deviation). Comparisons of means were made using the Student's test for matched samples and qualitative variables with the McNemar test. These tests made it possible to study the existence of statistical links between the factors considered and the AKN. Conditional logistic regression was used to identify the factors associated with AKN. Factors with a value of p < 0.20 in univariate analysis were taken into account for the multivariate model with a significance level of 5%. All analyses were performed using R^®^ software.

### Results

We recruited 303 consenting subjects comprising 101 patients with AKN and 202 controls. The mean patients age was 34.9 ± 10.7 years versus 35.6 ± 11.2 years for controls. The average duration of AKN progression in cases prior to consultation was 1831 days (5 years). The most frequently observed AKN lesions were papules (73/101; 72.3%), fibrous scars (42/101; 41.6%) and folliculitis/pustules (41/101; 40.6%) (Table 1). We found other lesions associated with AKN such as acne (29 cases; 28.7%), pubic folliculitis (13 cases; 12.9%) and folliculitis of the axillary hollows (4 cases; 4%). In terms of quality of life, the mean score of DLQI was 8.3 ± 5.2 (extremes: 0 to 22). Almost all patients (99/101; 98%) had an altered QOL. More than half of the patients (60/101; 59.5%) had mild to moderate changes in their QOL and more than a third (39/101; 38.6%) had significant or very significant changes in their QOL.

**Table 1:** Lesions observed in 101 patients

	Number	%
Papules	73	72.2
Fibrous scars	42	41.5
Folliculitis/pustules	41	40.6
Keloids scars	30	29.7
Ulceration	29	28.7
Nodules	21	20.8
Alopecia	12	11.9
Pus	9	8.9
Atrophic scars	7	6.9

In univariate analysis, having a BMI of 25 kg/m^2^ and above was associated with the risk of AKN (p < 0.001). Systolic hypertension (≥ 140 mmHg) was present in 23.8% of cases versus 7.9% of controls (p = 0.009). Diastolic hypertension (≥ 90 mmHg) was present in 33.7% of cases versus 29.2% of controls (p = 0.461) (Table 2).

**Table 2:** Comparisons of measured variables and calculation of odds ratio: in univariate analysis

Variables	Cases (n = 101)	Controls (n = 202)	p-value
Mean age (years) ± standard deviation	34.91 ± 10.7	35.62 ± 11.2	NC
Mean BMI (Kg/m^2^) ± standard deviation	27.0 ± 4.5	23.7 ± 2.9	< 0.001 T
Mean systolic blood pressure (mmHg) ± standard deviation	126.7 ± 22.50	115.7 ± 14.24	< 0.001^ T^
Mean diastolic blood pressure (mmHg) ± standard deviation	83.07 ± 15.86	75.74 ± 13.06	0.001^ T^
BMI (kg/m^2^), n (%)			
< 25	36 (35.6)	147 (72.8)	–
≥ 25	65 (64.4)	55 (27.2)	< 0.001^a^
Blood pressure, n (%)			
Systolic hypertension			
No	77 (76.2)	186 (92.1)	0.009^a^
Yes	24 (23.8)	16 (7.9)	
Diastolic hypertension			
No	67 (66.3)	143 (70.8)	0.461^a^
Yes	34 (33.7)	59 (29.2)	

NC: pairing variable of the two groups; BMI: body mass index; T: student test for paired samples; ^a^McNemar's test

In multivariate analysis, adjusted for other variables, having a BMI of 25 kg/m^2^ or more (OR = 4.91; p < 0.001) and having systolic hypertension (OR = 1.22; p = 0.010) were associated with AKN (Table 3).

**Table 3:** Association between AKN and hypertension in black subjects; conditional logistic model (N = 303)

	Multivariate analysis		
	aOR	95% for a OR	p-value
BMI (kg/m^2^)			
< 25	1	–	
≥ 25	4.91	[2.96–8.28]	< *0.001*
Systolic hypertension			
No	1	–	
Yes	1.22	[1.10–3.11]	*0.010*
Diastolic hypertension			
No	1	–	
Yes	0.84	[0.75–1.36]	0.574

### Discussion

The frequency of AKN and the difficulty of their management are arguments for researching the factors associated with these conditions, in order to prevent them if possible. It is a condition that so impairs the QOL of patients who suffer from it [3, 7], as in our study where almost all patients had an impairment in their QOL. We evaluated the association between hypertension and AKN in 101 patients compared to 202 matched controls. Our study is the first of its kind, to our knowledge, involving two groups matched with untreated patients in an African country where the prevalence of hypertension and AKN are relatively high. By matching our cases, we were able to avoid the biases of age and gender influence on blood pressure. We also searched for and excluded patients who had received therapies that could have influenced blood pressure such as intra-lesional corticosteroid injections.

In our study, not only were mean systolic (p < 0.001) and diastolic (p = 0.001) arterial pressures higher in cases than controls, the calculation of the odds ratio shows an association between systolic hypertension and the presence of AKN. Furthermore, the risk of AKN increases with BMI, as shown by the OR, which was 4.91 in overweight or obese subjects (BMI ≥ 25 kg/m^2^). Although high BMI is thought to be a mechanical risk factor for AKN (more folds in the occipital region, more risk of incarnation), it is a risk factor for high blood pressure. Our study therefore confirms that there is an association between AKN and systolic hypertension, as previously suggested by other authors [1, 4]. Although AKN and keloids do not have the same physiopathology, stage 3 of AKN leads to keloid formation according to the classification of Mahé et al. [8]. With regard to keloids, while some studies have found no association with hypertension [9], others have shown that hypertension is associated with their development, with both occurring more frequently in people of African descent than in Caucasians [10, 11]. Patients with keloid disorders are more likely to have concomitant hypertension than patients without keloid disorders [12]. There is an interaction between these factors that may be mediated by an alteration in the ratio of metalloproteinase-1 matrix to tissue inhibitor of metalloproteinase-1 (MMP-1/TIMP-1) that promotes the deposition of extracellular matrix. Endothelial dysfunction, which also occurs in high blood pressure, may also be involved in the pathogenesis of keloid disorders [13]. In addition, activation of the renin-angiotensin system is known to cause angiotensin II-induced arterial vasoconstriction resulting in elevated blood pressure. Similarly, the association between the renin-angiotensin system and tissue fibrosis is well documented [14, 15]. The mechanism highlighting the association between renin-angiotensin system activity and fibrosis is thought to be an increased expression of growth factor (TGF-1) caused by angiotensin II, which plays a central role in fibrogenesis [16]. This role of angiotensin II in both hypertension and fibrogenesis may also partly explain the significant association between hypertension and AKN. For example, improvement of keloid lesions in hypertensive patients has been observed after treatment with antihypertensive drugs, such as calcium channel blockers and angiotensin converting enzyme inhibitors [17, 18]. Howerver, we didn’t find a pathogenic explanation between AKN without keloids (stages 1, 2) and hypertension.

### Limitations of study

The main limitation of this study is that, we have not evaluate the association between, AKN severity and hypertension, because of the number of patients. Ideally, the link between the severity of AKN and hypertension should be assessed. A multicentre study to have a larger number of cases will be necessary to look for this link between AKN severity and hypertension. Secondly, we did not assess the quality of life in the controls to make a comparative study on this point.

## Supplementary information

**Additional file 1.** Questionnaire used for general and dermatological examination in this study.

## Data Availability

All data generated or analysed during this study are included in this published article.

## Abbreviations

AKN: acne keloidalis nuchae
BMI: body mass index
DLQI: dermatology life quality index
MMP: matrix metalloprotease
QOL: quality of life
aOR: adjusted odds-ratio
TIMP: tissue inhibitors of metalloproteinases
TGF: transforming growth factor
