# Supplementary material for: Acne keloidalis nuchae and hypertension in black subjects: a case–control study
Source: BMC Res Notes. 2020 Sep 14;13:431. doi: 10.1186/s13104-020-05274-0 (PMC7491137; doi:10.1186/s13104-020-05274-0)
Supplement: Supplementary file 1 — Additional file 1. Questionnaire used for general and dermatological examination in this study. [file 13104_2020_5274_MOESM1_ESM.docx]

**Questionnaire**

Registration Number: [__][__][__][__][__]

**I- Sociodemographic informations and general examination**

Age of the patient (years) : [__][__]

Sex : Male [_] Female [_]

Weight (kg) : [__][__][__]

Height (m) : [__][__][__]

Body mass index: $\frac{Weight (kg)}{{(Height, m)}^{2}}$ =

Blood pressure measurement, first visit (mmHg) : Left hand…….. Right hand…….

Blood pressure measurement, second visit (mmHg) : Left hand…….. Right hand…….

**II- Antecedents (medical history)**

Hypertension : Yes [_] No [_]

Obesity : Yes [_] No [_]

**III- Clinical signs**

What types of lesions are present?

Papules [_] Folliculitis/Pustules [_]

Ulceration(s) [_] Pus [_]

Nodules [_] Alopecia [_]

Fibrous scars [_] Atrophic scars [_]

Keloid scars [_]

**Functionnal signs** : Absent [_]; Pain [_]; Pruritus [_];

Bleeding [_]; Oozing [_] ; Stretching [_]

**Duration of lesions evolution (to be specified)** : _ _ _ _ __ _ _ _ _ _ _ _ _ _ _ _ _ _ _ _ _ _

**Stadification**

Stage 1 [_]; Stage 2 [_] ; stage 3 [_]

**IV- Lesions associated with acne keloidalis nuchae**

Acne Yes [_] No [_]

Pubic folliculitis Yes [_] No [_]

Folliculitis of the axillary hollows Yes [_] No [_]

Others Yes [_] No [_], if yes, please specify
